# Supplementary material for: Hypovascular tumors developed into hepatocellular carcinoma at a high rate despite the elimination of hepatitis C virus by direct-acting antivirals
Source: PLoS One. 2020 Aug 13;15(8):e0237475. doi: 10.1371/journal.pone.0237475 (PMC7425876; doi:10.1371/journal.pone.0237475)
Supplement: S1 Table — (DOCX) [file pone.0237475.s001.docx]

S1 Table: The baseline characteristics of patients and rate of HCC development for each survey method

|  | **US only**  **(n=227)** | **CT or MRI (n=168)** | ***P* value** |
| --- | --- | --- | --- |
| Age, years | 68.2±9.3 | 68.9±9.4 | 0.464 |
| Male, n (%) | 91 (40.1) | 76 (45.2) | 0.178 |
| Liver Cirrhosis, n (%) | 26 (11.5) | 61 (36.3) | <0.001 |
| History of interferon-based therapy, n (%) (n=394) | 108 (47.6) | 92 (54.8) | 0.270 |
| History of HCC therapy, n (%) | 5 (2.2) | 29 (17.3) | <0.001 |
| Platelets, ×10^4^/μL | 15.3±5.3 | 12.3±5.1 | <0.001 |
| Total bilirubin, mg/dL | 0.8±0.3 | 0.9±0.4 | 0.016 |
| ALT, U/L | 48±41 | 50±35 | 0.151 |
| GGT, U/L | 41±35 | 44±39 | 0.041 |
| Albumin, g/dL (n=385) | 4.0±0.4 | 3.9±0.5 | <0.001 |
| Hyaluronic acid, ng/mL (n=340) | 169±385 | 285±424 | <0.001 |
| Fib-4 index | 3.64±2.34 | 5.55±3.94 | <0.001 |
| AFP (Before), ng/mL (n=385) | 8.3±16.4 | 13.6±28.2 | <0.001 |
| AFP (End of treatment), ng/mL (n=373) | 5.5±15.2 | 5.2±6.4 | 0.016 |
| DCP, mAU/mL (n=302) | 20.5±9.6 | 21.8±11.4 | 0.514 |
| HCC development n (%) | 9 (4.0) | 27 (16.1) | <0.001 |

Data are shown as the mean ± standard deviation.

US, ultrasound sonography; HCC, hepatocellular carcinoma; ALT, alanine transaminase; GGT, γ-glutamyltransferase; AFP, α-fetoprotein; DCP, des-γ-carboxy prothrombin; SVR, sustained virologic response
