# Supplementary material for: Hypovascular tumors developed into hepatocellular carcinoma at a high rate despite the elimination of hepatitis C virus by direct-acting antivirals
Source: PLoS One. 2020 Aug 13;15(8):e0237475. doi: 10.1371/journal.pone.0237475 (PMC7425876; doi:10.1371/journal.pone.0237475)
Supplement: Attachment — Submitted filename: Response to Reviewers.docx [file pone.0237475.s004.docx]

Thank you for your valuable feedback. We have revised the manuscript based on the comments.

Reviewer #1

1. *It is recommended to give a definition of how many millimeter or more the growth of hypovascular tumors should be.*

Thank you for your comments. HCC development was defined by 20% growth or hypervascularization of a hypovascular nodule. We have added this to the Materials and Methods section.

2. *The pixels in Figures are a little bit coarse.*

We have improved the resolution of the figure.

Reviewer #2:

1. *Please show whether the survey methods (CT/MRI vs echo) before treatment affect incidence of HCC or not, and please described when this survey was conducted (all patients were surveyed at initiation point of DAA?)*

The patients who underwent CT or MRI before DAA therapy had higher HCC development rates than those who underwent US alone. Because the patients who underwent CT or MRI more frequently had liver cirrhosis and a history of HCC therapy. We have created S1 Table and described the following in the Results section.

The patients who underwent CT or MRI before DAA therapy had higher rates of HCC development than those who underwent US alone. Because the patients who underwent CT or MRI more frequently had liver cirrhosis and a history of HCC therapy, had higher total bilirubin, GGT, hyaluronic acid, Fib-4 index, and AFP values before therapy and at the end of treatment, and had lower platelet counts and albumin levels than those who underwent US alone (S1 Table).

This study was a prospective observational study conducted in 29 patients with hypovascular tumors that were confirmed by CE-MRI or CT. Informed consent was obtained after the confirmation. Some of the patients applied for a medical subsidy program in Japan after giving their consent. Therefore, most patients were surveyed before DAA therapy. These surveys were conducted approximately 50 days before therapy.

We have described this in the Materials and Methods section. In addition, 452 patients received DAA therapy after they were confirmed to be tumor-free by US, CT, or MRI. However, we did not confirm the exact US survey points before therapy. Thus, the initiation of the observation period was defined as the initiation of DAA treatment. We have described this as a limitation of the present study.

2. *In Materials, and Method, Patients; the authors described that “ 168 without a hypovascular tumor underwent CE-MRI or CT within 2 year before therapy or during DAA therapy”
This reviewer think that 2 years is too long for diagnosis of no hypovascular tumor at baseline. To avoid this, please limit the patients who were conducted CT around the point of initiation of DAAs, or to described this in limitation of this study.*

Thank you for your comments. I agree with the reviewer's suggestions. Unfortunately, there were few patients who underwent CT or MRI immediately before DAA therapy. Since it overlaps with the comments in 1, we have described the following as a limitation of this study.

Fourth, 29 patients had hypovascular tumors that were confirmed by CE-MRI or CT. These surveys were conducted approximately 50 days before therapy. Further, 452 patients received DAA therapy after they were confirmed to be tumor-free by US, CT, or MRI. However, we did not confirm the exact US survey points before therapy. Thus, the start of the observation period was defined as the time at which DAA treatment was initiated. In addition, 168 patients without a hypovascular tumor underwent CE-MRI or CT within 2 years before therapy or during DAA therapy. Unfortunately, there were few patients who underwent CT or MRI immediately before DAA therapy. We cannot rule out the possibility of growth or hypervascularization of hypovascular tumors, or HCC development in patients who were tumor-free before DAA therapy. Thus, a future study should analyze a larger cohort which imaging surveillance was performed immediately before DAA therapy.

3. *In figure2, please show the analysis in patients who achieved SVR only.*

We have shown the analysis in patients who achieved an SVR only in Figure 2 and 3. We therefore corrected the data in the Abstract, Results and Discussion.
